# A Novel Artificial Bee Colony Approach of Live Virtual Machine Migration Policy Using Bayes Theorem

**DOI:** 10.1155/2013/369209

**Published:** 2013-12-09

**Authors:** Gaochao Xu, Yan Ding, Jia Zhao, Liang Hu, Xiaodong Fu

**Affiliations:** ^1^College of Computer Science and Technology, Jilin University, Changchun, Jilin 130000, China; ^2^Key Laboratory of Symbolic Computation and Knowledge Engineering of Ministry of Education, Jilin University, Changchun, Jilin 130000, China

## Abstract

Green cloud data center has become a research hotspot of virtualized cloud computing architecture. Since live virtual machine (VM) migration technology is widely used and studied in cloud computing, we have focused on the VM placement selection of live migration for power saving. We present a novel heuristic approach which is called PS-ABC. Its algorithm includes two parts. One is that it combines the artificial bee colony (ABC) idea with the uniform random initialization idea, the binary search idea, and Boltzmann selection policy to achieve an improved ABC-based approach with better global exploration's ability and local exploitation's ability. The other one is that it uses the Bayes theorem to further optimize the improved ABC-based process to faster get the final optimal solution. As a result, the whole approach achieves a longer-term efficient optimization for power saving. The experimental results demonstrate that PS-ABC evidently reduces the total incremental power consumption and better protects the performance of VM running and migrating compared with the existing research. It makes the result of live VM migration more high-effective and meaningful.

## 1. Introduction

VM technology [1, 2], one of the most important technologies in cloud computing, is not only a way to implementing cloud computing such as Infrastructure as a Service (IaaS) [3] architecture but also for the embody of the cloud computing idea, whereas live VM migration technology, which is widely used for the maintenance management in virtualized cloud computing data centers, is the representative of the VM technologies. When a VM needs migrating from source host to target host for some goal or several goals, generally the migration target of a VM is chosen randomly as long as the host can accommodate it and then one can automatically or manually move the VM to a target host. It is obvious that the way to randomly choose a target host for a live VM migration which some event has aroused and has more than one available target host meeting the requirements of that event is not efficient in all respects.

Nowadays, power consumption of data centers has huge impact on environments. Researchers have been seeking to find effective solutions to minimize power consumption of data centers while keeping the desired quality of service. On the background of low-carbon world and cloud computing era, researchers have proposed the field of green cloud computing based on cloud computing and virtualization. It aims at reducing power consumption in cloud computing data centers. In this paper, we focus on live VM migration policy based on green data center. In a cloud data center, there are always some VMs needing to be migrated for some reasons. Generally speaking, the migrant VM has many available target hosts. However, only one target host is most suitable for the VM in order to minimize the total incremental power consumption in cloud data center. To achieve green cloud data center, in the search direction of VM provisioning the problem is similar to that of live VM migration. Both are to find out the optimal target host.

Many papers have presented some heuristic approaches to find optimal solutions aiming to minimize power consumption. The basic idea is that according to the current situation and history of a cloud data center, the controllers have searched for a best policy by using their proposed approaches. The problems of convergence and local optimization have been challenging the research direction. On the other hand, we know that a data center does not have abilities in predicting the size and type of the next workloads. Therefore, the optimal policy which the proposed approaches have found out in a short-term is not necessarily the optimal solution in a long-term. In a word, the global best which of some VM the proposed approaches have found out in an algorithm cycle may be a local best in a long-term process. Thus, the probability methods and ideas should be introduced into these heuristic approaches aiming at this kind of problems. Besides, as the capability that the current random migration policy and optimal migration policy adapt to a dynamic cloud environment is not excellent enough, they may cause many failure events of live VM migration in a real and dynamic cloud environment. To address these problems, this paper presents a novel heuristic approach PS-ABC, which designs and employs the improved artificial bee colony approache and utilizes the binary search idea and the Boltzmann selection policy to achieve a more efficient power saving optimization. Also, the Bayes theorem is introduced into the proposed PS-ABC approach and makes it have capability in fast converging to global optimal solution while improving the performance of live VM migration. That is, the failure rate of VM migration is decreased. Compared to the random approach and other existing optimization approaches, the proposed PS-ABC has reduced more power consumption and failure events of live VM migration to contribute to achieving better green cloud data centers.

The rest of the paper is organized as follows. In Section 2, we present the related work related to our proposed approach aiming to green cloud data center and the reasonable prerequisites are shown clearly. In Section 3, the analysis of the problem proposed in this paper and its formulation are given. In Section 4, the algorithm and implementation of PS-ABC are introduced in detail. In Section 5, the experimental results and analysis on CloudSim platform are given. Finally, in Section 6, we summarize the full paper and future work is put forward.

## 2. Related Work

As far as we know, the proposed problem which refers to finding a fit target host for a live VM migration according to the standard of minimizing the increment power consumption has not been widely researched in the related fields. However, most researchers have focused on some problems which are similar to the proposed problem in this paper. Also, some researchers have focused on the direction that aims to other problems of cloud computing and also to minimize the incremental power consumption but utilizes the technology of live VM migration. Similarly, there are also some researchers studying the direction which utilizes live VM migration to move these VMs in order to fulfill the requirement of performance and workload limitation while minimizing the power drawn by a cloud data center. In fact, most problems of them are just to find an optimal host for each VM, which will be migrated or be created, under the target of minimizing the power drawn by a cloud data center. Thus, the related work of the kind of problems of green cloud data centers will be discussed briefly in this section.

Rusu et al. in [4] have presented a cluster-wide QoS-aware technique that dynamically reconfigures the cluster to reduce power consumption during periods of reduced load. The proposed system consists of two important components, namely, front end manager and a local manager. While the front end manager finds the servers which should be turned on or off in terms of a given system load, the local manager will utilize Dynamic Voltage and Frequency Scaling (DVFS) technique to conserve power. The main shortage of the approach is the on/off policy. It relies on the table of values and needs computing offline. However, the system does not make use of server consolidation through VM migration and thus its on/off policy may not be much effective.

Srikantaiah et al. [5] have investigated the problem of dynamic consolidation of applications serving small stateless requests in data centers to minimize the power consumption. They modeled the problem as a multidimensional bin packing problem. However, the proposed model does not describe the degradation of performance due to the consolidation. Besides, the power drawn may rely on a particular set of applications combined on a computer node. A heuristic for the defined bin packing problem is proposed by the authors. The heuristic is based upon the idea of minimizing the sum of the current allocations' Euclidean distances to the optimal point at each server. The application workload will be allocated to a server using the proposed heuristic since a request to execute a new application is received. Without the sufficient capacity of all active, the system will switch on a new server while reallocating all the applications using the same heuristic in an arbitrary order. The proposed approached is fit for heterogeneous environments; however, it has several shortcomings. First, the approach assumes that all applications' resource requirements are known in advance and constant. Second, performance and power overhead, which the authors do not take into account, is caused by migration of state-full applications between nodes. The frequent switching servers on/off also generate significant costs which are not negligible for a real-world system.

Verma et al. [6] have contributed power and migration cost-aware application placement by exploiting the power management capabilities of virtualization. The authors have designed a new application (virtual machines) placement architecture called pMapper. It consists of three major parts, namely, a performance manager to dynamically resize the VM, a power manager for CPU throttling, and a migration manager to identify the target host for migration using a knowledge base. They have expounded that for power-aware scheduling approaches, estimates of power values are not required, and only if the scheduling algorithm has abilities in finding out which server minimizes the incremental increase in total power owing to the new VM being placed, it can place the given VM to appropriate host. In pMapper, two algorithms are implemented. One is First Fit Decreasing (FFD) by which more power-efficient servers are utilized first without balancing the load. The other is incremental First Fit Decreasing (iFFD) which considers the fixed target utilization of each server and achieves server consolidation by live VM migration. The proposed pMapper architecture minimizes power and migration costs with ensuring the performance. Our approach is based on a heuristic approach which exploits the concept of minimizing total increase in the incremental power due to the new VM migrations. The proposed architecture is simple and does not need any knowledge base to achieve significant reduction in the power consumption.

Li et al. in [7] have proposed an approach named EnaCloud, which enables application live placement dynamically with consideration of power efficiency in a cloud platform. In EnaCloud, they use a virtual machine to encapsulate the application, which supports applications scheduling and live migration to minimize the number of running machines, so as to save power. Specially, the application placement is abstracted as a bin packing problem, and a power-aware heuristic algorithm is proposed to get an appropriate solution. In addition, an over-provision approach is presented to deal with the varying resource demands of applications. However, the over-provision approach has risk to optimize this problem. It may cause more cost in order to reduce less cost with a certain probability.

Jeyarani et al. [8] have proposed self-adaptive particle swarm optimization (SAPSO) for efficient virtual machine provisioning in cloud aimed at the following: when mapping a set of VM instances onto a set of servers from a dynamic resource pool, the total incremental power drawn upon the mapping is minimal and does not compromise the performance objectives. The advantage of the proposed solution is obvious. It has focused on not only improving the performance of workload facilitating the cloud consumers but also developing the power efficient data center management to facilitate cloud providers. However, the approach still may be inefficient and cause some additional events and costs from a long-term perspective as it does not take the future workload into account. Our proposed algorithm PS-ABC is a heuristic approach which is based on ABC, one of swarm intelligence algorithms and introduces the Boltzmann selection idea into it.

In this paper, PS-ABC has a prerequisite. We know that PS-ABC is to find the target host of each VM from all *m* hosts for the *n* migrant VMs. We assume that each VM's target host found by PS-ABC will not be the host which the VM is moved out from. The approach provides service to live VM migration aiming to green cloud data center. Thus, the fact that the VM should be moved out for some reason is the premise of our approach. Talking objectively, the prerequisite is justified from a certain perspective. Since a VM needs to be migrated from its source host, its candidate hosts will not include its source host. Otherwise, it does not need a migration event. Further, this matter that a host both needs move out VMs and has ability in receiving VMs within a time window Δ*t* is impossible and nonobjective. It can be seen that for all the migrant VMs, the hosts each of which is the source host of some migrant VM will not be the target hosts. Therefore, the proposed prerequisite is reasonable and does not impact the performance and efficiency of our approach.

## 3. The Proposed Problem and Its Formulation

### 3.1. The Proposed Problem

In IaaS cloud platform, some of the running VMs may be required to be migrated for some reasons. Generally, the target of live VM migration is more than one host. Moreover, the different selections of target hosts will cause the different power consumption. Therefore, a high-efficient power saving placement selection policy to migrate the migrant VMs onto the right fit hosts is necessary.

### 3.2. Problem Formulation

We now formulate the problem of migrating *n* VMs onto *m* hosts. Its solution can be represented by an *n* dimension of solution vector, each element of which denotes the target host of the migrant VM which its location represents. We assume that there are *m* available hosts in the resource pool and the hosts are heterogeneous and dynamic while using space shared allocation policy. The hosts change their state dynamically according to the load. The problem can be stated as follows. Find a VM-host set Vh of placement selections, such that the total incremental power consumption caused by the migrated VMs onto hosts is minimized, while maximizing the performance by fulfilling the resource requirements of maximum number of VMs. We define a four tuple *S* = {PH, VM, PC, Vh} for our problem scenario. PH is a set of *m* available physical hosts denoted by PH(*m*, *t*) = {PH_1_, PH_2_, PH_3_ …, PH_*m*_}, available at migrating start time *t*. VM is a set of *n* VMs denoted by VM (*n*, *t*, Δ*t*) = {VM_1_, VM_2_, VM_3_,…, VM_*n*_} accumulated within a time window Δ*t*. PC(*m*, *t*) = {PC_1_, PC_2_,…, PC_*m*_} is the power consumption by the *m* physical hosts in resource pool. The problem is multimodal, having more than one placement selection which meets the performance constraints of the VM requests. Therefore, our goal is to find all of *o* candidate placement selections which maximize the performance and then to find which one minimizes the power consumption among them. To fulfill performance constraints, a metric denoted by *η*_*r*_ representing resource fulfillment requirement is defined as follows:

(1)    ηr=∑i=1n∑j=1mϕij, i∈{1,2,3,…,n},j∈{1,2,3,…,m}, r∈{1,2,3,…,s},

where *ϕ*_*i*_^*j*^ denotes the placement selection of *i*th VM on the *j*th host and is defined as follows. *s* denotes the total number of placement selections.

Consider

(2) $$\begin{array}{c} \phi_{i}^{j}=\{\begin{array}{cc} 1 & \\ \;\text{if}\;{\text{VM}}_{i}\;\text{has}\;\text{been}\;\text{allocated}\;\text{to} & \\ \;{\text{Host}}_{j}\;\text{and}\;rcr{\text{VM}}_{i}\leq acr{\text{Host}}_{j}, & \\ \;i\in \left\{1,2,3,\ldots ,n\right\} & \\ 0 & \\ \;\text{if}\;{\text{VM}}_{i}\;\text{has}\;\text{been}\;\text{allocated}\;\text{to} & \\ \;{\text{Host}}_{j}\;\text{and}\;rcr{\text{VM}}_{i}>acr{\text{Host}}_{j}, & \\ \;j\in \left\{1,2,3,\ldots ,m\right\} & \\ \text{invalid} & \\ \;\text{if}\;{\text{VM}}_{i}\;\text{has}\;\text{not}\;\text{been}\;\text{allocated}\;\text{to} & \\ {\;\text{Host}}_{j}. & \end{array} \end{array}$$

In (2), *rcr*VM_*i*_ denotes the minimum computing resource requirements of *i*th VM and *acr*Host_*j*_ denotes the available computing resource of *j*th host. Out of *s* placement selections, *o* placement selections with maximum *η*_*r*_ values give the best performance and they are represented as Vh[*m*, *n*, *o*, *t*].

In fact, Vh[*m*, *n*, *o*, *t*] represents *o* solution vectors with maximum *η*_*r*_ values. The second metric is based on the power consumption. The migration of successive VMs is represented as Vh[*m*, *n*, *r*, *t* + *t*_0_(*k*)] where *r* represents any one of *o* placement selections and *k* is an integer increasing with successive migrating, representing a stage. We can understand like that if *k* is 3, the approach will migrate the third VM to the host which is denoted at the third location of the *r* vector. The *o* placement selections at stage *k* is represented as Vh[*m*, *n*, *r*, *t* + *t*_0_(*k*)]*r* ∈ {1,2, 3,…, *o*} and their corresponding power consumption is represented as *ξ*_*k*_^*r*^. Its meaning is that, according to *r* placement vector after the system has migrated the *k*th VM to its target host, the total power consumption by the cloud data center is *ξ*_*k*_^*r*^. The meaning of *ξ*_*k*−1_^*r*^ can be imaged. In this paper, these parameters of *ξ*_*k*_^*r*^ can be obtained by using simulation platform in the experiment. Now the incremental power consumption due to migrating Vh[*m*, *n*, *r*, *t* + *t*_0_(*k*)] with respect to previous migration stage Vh[*m*, *n*, *r*, *t* + *t*_0_(*k* − 1)] is defined by

(3) $$\begin{array}{c} \Delta P=\left(\xi_{k}^{r}\right)-\left(\xi_{k-1}^{r}\right),\;r\in \left\{1,2,3,\ldots ,o\right\}. \end{array}$$

For better power saving, the following *δP* is minimized to get the optimal solution and it is denoted as follows:

(4) $$\begin{array}{c} \delta P=\overset{n}{\underset{k=1}{\sum }}\left(\left(\xi_{k}^{r}\right)-\left(\xi_{k-1}^{r}\right)\right),\;r\in \left\{1,2,3,\ldots ,o\right\}. \end{array}$$

Therefore, the proposed approach maximizes *η*_*r*_ for better performance requirements and then minimizes *δP* for power efficiency.

## 4. Heuristic Methodology for Efficient Placement Selections of Live VM Migration

### 4.1. The Proposed System Architecture

**Figure 1:**
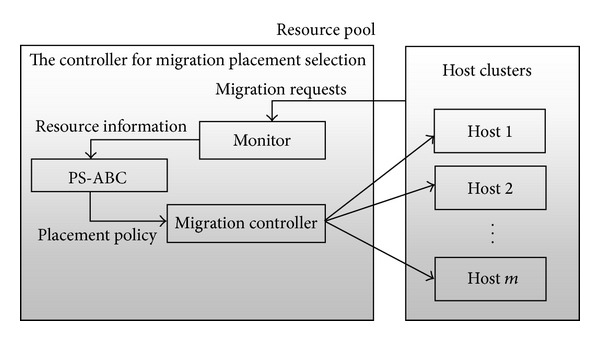
The view of PS-ABC's architecture.

Figure 1 depicts the proposed architecture for cloud environment. It shows the position of the controller PS-ABC for migration placement selections and its interaction with other entities [9, 10]. In a time window Δ*t*, the Monitor gets the requests of live VM migration and is updated with the available number of computing resource such as CPUs, memory, and storage as well as power consumption. At the end of Δ*t*, the Monitor transfers the information to the controller. The controller generates the placement policy by using the proposed approach and obtained information. Then, it transfers the policy to the migration controller which controls and executes live migration of the VMs. The VMs are moved onto their target hosts eventually.

### 4.2. Solution Representation

In order to design an efficient ABC-based approach for finding the optimal solution vector of the target hosts of all migrant VMs in a time window Δ*t*, the primary problem is the solution representation as it represents a direct relationship between the problem domain and the food sources in ABC. During the applying of a ABC-based approach, we know that a food source is denoted as a solution of the specific problem. Here, there are *n* VMs to be migrated into *m* physical hosts. So, the proposed problem is an *n* dimensional problem, leading to the fact that the food sources are represented as *n* dimensional solution vectors. Each dimension has a discrete set of possible placement selection limited to *m*. A solution vector called a food source is denoted by *x*_*i*_^*k*^ = (*x*_*i*1_^*k*^, *x*_*i*2_^*k*^,…, *x*_*ij*_^*k*^,…, *x*_*iD*_^*k*^), where the *x*_*ij*_^*k*^ value represents the Number of target host of the *j*th migrant VM of the *i*th possible solution vector in *k*th generation.

### 4.3. The Algorithm of PS-ABC

The proposed PS-ABC approach is a novel heuristic live VM migration policy, which is used for the target location selection of live VM migration. It is based on the improved ABC approach, in which firstly we present the uniform random initialization idea to improve the initialization process of food sources and thus to make the PS-ABC approach have a better global search ability at the very beginning; secondly, we utilize the binary search idea to improve the expression, by using which each employed bee produces a candidate food position, for increasing the neighborhood searching efficiency of the employed bees and thus to achieve the high-efficient global search and local convergence; thirdly the PS-ABC approach employs the Boltzmann selection policy to achieve the probability selection of onlookers and thus to avoid prematurity and make the PS-ABC approach have the self-adaptive selective pressure adjustment so as to achieve having the better global exploration ability in the early stage and the better exploitation ability in the later stage; finally we introduce the Bayes Theorem into the proposed PS-ABC approach to optimize its convergence efficiency and improve its accuracy.

### 4.4. The Implementation of PS-ABC

In this section, we describe the specific process of PS-ABC. Details are as follows.

#### 4.4.1. Monitor the Requests of Live VM Migration

The proposed controller gets periodically the requests of live VM migration and the information of computing resource. The time is denoted as Δ*t*. In other words, the proposed PS-ABC is executed once at intervals of Δ*t*. In a time window Δ*t*, the number of VM migration requests obtained is the dimension number of a solution vector of the problem to be resolved by the proposed approach. After a Δ*t* ends, the monitor transmits the relevant information required by the proposed approach to the module used for running the algorithm.

#### 4.4.2. Initialize the Parameters and Food Sources

The PS-ABC module receives all required information and begins running the algorithm. First, the parameters required by the approach are initialized such as the number FSN of food sources. For each food source, there is only one employed bee. That is, the number of employed bees is equal to FSN. In ABC, the number of onlookers is equal to the number FSN of employed bees. The maximum number of iterations is MNI. The dimension of the problem to be solved is *D*. The controlling parameter for the ABC process is LIMIT. The parameter LIMIT is used to control when an employed bee abandons its current food source and becomes a scout to have a new random candidate food source. In the proposed PS-ABC approach, the parameter LIMIT does not only play the role but also is taken use of to control when the Bayes theorem works for achieving fast convergence of the algorithm. After initializing the parameters, the PS-ABC begins to generate the initial population of FSN food sources. In the PS-ABC, the initial food sources are not produced at random within the search space but are generated in terms of the proposed uniform random idea. The whole search space is evenly divided into FSN small subspaces. Within each subspace, a food source is generated randomly. As a result, the FSN initial food sources are generated in the whole search space. In this way, the PS-ABC approach has a better global search potential while retaining the traditional randomness. Further, in this case where the added overhead is almost negligible, the performance improvement is noticeable due to the employment of uniform random initialization idea, not only from the perspective of diversity and global search optimization potential but also from the perspective of improving the search efficiency and achieving fast convergence.

#### 4.4.3. Main Iterative Process of the PS-ABC Algorithm

After the swarm of FSN employed bees, the swarm of FSN onlookers, the initial population of FSN food sources, and all the relevant parameters are initialized, the PS-ABC begins to perform the first round of iterations. Obviously, each food source has an employed bee. The FSN employed bees concurrently calculate the first fitness value *η*_*r*_ of the FSN initial food sources on performance requirements according to the expression, (1) and (2). Then they calculate the second fitness value *δP* of the FSN initial food sources on power consumption and each employed bee keeps its two fitness values in its own memory. Afterwards, these employed bees perform neighbourhood search according the following expression:

(5) $$\begin{array}{c} \nu_{ij}=\frac{x_{ij}+x_{kj}}{2}, \end{array}$$

where *k* ∈ {1, 2, …, FSN} and *j* ∈ {1, 2, …, *D*} are randomly chosen indexes. *x*_*ij*_ is the current food source which the *i*th employed bee is located at. Although *k* is determined randomly, it has to be different from *i*. In the classic ABC approach, the expression for the employed bees' neighbourhood search is as follows:

(6) $$\begin{array}{c} \nu_{ij}=x_{ij}+\phi_{ij}\left(x_{ij}-x_{kj}\right), \end{array}$$

where *ϕ*_*ij*_ is a random number between −1 and 1. It controls the production of a neighbour food source position around *x*_*ij*_ and the modification represents the comparison of the neighbour food positions visually by the bee [11, 12]. In the proposed PS-ABC approach, the parameter *ϕ*_*ij*_ is not a random number and is set as −1/2 to utilize the binary search and thus to achieve more efficient neighbour search. It also makes the PS-ABC approach have the better exploration and exploitation abilities at the same time to some extent. In our proposed problem, the performance fulfillment is the constraint condition. As a result, the first fitness value *η*_*r*_ should be maximized even if the current situation cannot make *η*_*r*_ equal to *D*. Under this background, the PS-ABC approach gives each employed bee *AL* chances to attempt to find the new food source which can make *η*_*r*_ equal to *D* during neighbourhood search. *AL* is a predetermined controlling parameter in the proposed PS-ABC approach. After that, if an employed bee still cannot find that kind of food source, the employed bee will return to the food source which has the maximum *η*_*r*_ value during *AL *times of neighbourhood search. That is, the result of neighborhood search of that employed bee is the food source which has the maximum *η*_*r*_ value during *AL* times of neighbourhood search. Each employed bee compares the new candidate food source by neighborhood search process with the current food source in the memory. If the *η*_*r*_ value of the new food source is larger than that of the old source, it is replaced with the old one in the memory. If the new food source has smaller *η*_*r*_ value than the old source, the old one is retained. If the new food source has equal *η*_*r*_ value as the old source, the second fitness value *δP* will be compared. If the new food source has equal or smaller *δP* value than the old source, it is replaced with the old one in the memory. Otherwise, the old one is retained. Now we define a partial order: ≺_*η*_*r*_,*δP*_ as follows:

(7) xα≺ηr,δPxβ if  (xα·ηr>xβ·ηr) or  ((xα·ηr=xβ·ηr),(xα·δP<xβ·δP)).

It represents that the food source *x*_*α*_ is better than the food source *x*_*β*_. After neighborhood search process of the FSN employed bees in the first round of iterations, the PS-ABC approach will compare the first fitness *η*_*r*_ values of all employed bees' food sources. The food sources whose *η*_*r*_ values are not the maximum of all set their *δP* values to 0.

All these employed bees share their information with onlookers by dancing within the hive and then the onlookers select one of the food sources. The preference of a food source by an onlooker bee depends on the second fitness value *δP* of that food source. As the second fitness value *δP* of the food source decreases, the probability with the preferred source by an onlooker bee increases. In order to dynamically adjust selection pressure during searching the optimal solution, the PS-ABC approach does not utilize the simple roulette wheel selection but introduces the Boltzmann selection policy into the searching process to achieve the selection probability with which onlooker bees choose their food sources to follow. According to Boltzmann selection policy, the probability with the food source located at *x*_*i*_ which will be chosen by an onlooker bee can be expressed as

(8) $$\begin{array}{c} p_{i}=\frac{\exp\left(\mu_{i}/T\right)}{\sum_{n=1}^{\text{FSN}}\exp\left(\mu_{n}/T\right)},\\ T=T_{0},\;\;c=1,\\ T=\left(\alpha^{c-1}\right)T,\;1<c\leq \text{MNI}, \end{array}$$

where *T* is the current temperature, *T*_0_ is the initial temperature, *α* is a number between 0 and 1, *c* is the current number of iterations of PS-ABC, and *μ*_*i*_ is the reciprocal of the *i*th food source's second fitness value. That is,

(9) $$\begin{array}{c} \mu_{i}=\frac{1}{\delta P_{i}}. \end{array}$$

In order to calculate the probability value *p*_*i*_, we do not directly utilize the fitness value *δP*_*i*_ of the *i*th food source but take use of its reciprocal. This is because our proposed problem needs to make the food source with a less power consumption have a larger probability value to be used for onlooker bees' selection. The reasonable transformation is simple and necessary.

After watching the dances of employed bees, an onlooker bee goes to the region of the food source located at *x*_*i*_ by the probability *p*_*i*_ and determines a neighbourhood food source to take its nectar depending on the fitness values. In other words, the onlooker bee selects one of the food sources after making a comparison among the food sources around *x*_*i*_. The position of the selected neighbourhood food source is calculated as expression (6). After the candidate food source position *ν*_*ij*_ is produced and then evaluated by the onlooker bee, its performance is compared with that of *x*_*ij*_. If *ν*_*i*_≺_*η*_*r*_,*δP*_ *x*_*i*_, then the position *x*_*i*_ of the food source is changed to be *ν*_*i*_; otherwise *x*_*i*_ is kept as it is.

**Figure 2:**
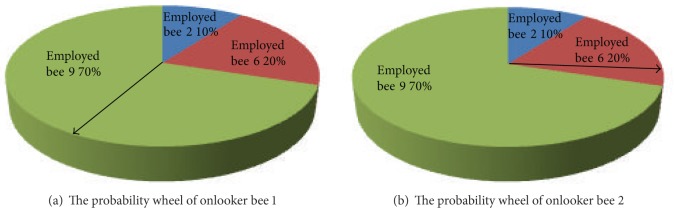
Examples of the probability wheel for onlooker bees.

After each of all onlooker bees of each employed bee finishes neighbourhood search, the optimal food source so far is recorded in the whole search space. The first round of iterations ends. The iteration number increases by 1. The PS-ABC approach determines whether there exists any food source which has not improved for LIMIT rounds of iterations continuously and the food source is not the best one during LIMIT rounds of iterations. If yes, the food source is abandoned. And its employed bee becomes a scout. The scout will randomly search for a new food source within the whole search space according to the following expression:

(10) $$\begin{array}{c} x_{ij}=x_{j}^{\min}+r\left(x_{j}^{\max}-x_{j}^{\min}\right), \end{array}$$

where *x*_*j*_^min⁡^ and *x*_*j*_^max⁡^, respectively, represent the minimum and maximum of the *j*th dimension of all available food sources in the whole search space. *r* is a random number between 0 and 1. The approach checks whether the maximum iterations number is reached. If yes, the PS-ABC approach ends and returns the global optimal solution. Otherwise, the approach begins to perform the next round of iterations. That is, all the employed bees begin neighbourhood search according to the expression (5). As described above, after a neighbourhood search if the fitness value *η*_*r*_ of an employed bee's new candidate food source is not *D*, the employed bee can continue to have neighbourhood search and have *AL* chances. If *AL *times of neighbourhood search still cannot make it find a new food source with *η*_*r*_ equal to *D*, its result of neighbourhood search is the best one of the *AL *candidate food sources. If it is better than the old one, the food source position of each employed bee is updated to the result of neighbourhood search in the memory. Otherwise, the old one is kept. Then, the *η*_*r*_ values of all employed bees are compared to get the largest ones. The fitness value *δP* of each of the food sources with the largest *η*_*r*_ values is calculated by its employed bee according to expressions (3) and (4). The *δP* values of other food sources are set to 0. For onlooker bees' selection, the probability *p*_*i*_ values of all food sources are calculated according to expressions (8) and (9). Each of the FSN onlooker bees reselects its employed bee to follow by using the probability wheel as shown in Figure 2.

After an onlooker bee reaches its employed bee's food source, it begins to perform neighborhood search according to expression (6). The new candidate food source *ν*_*i*_ of an onlooker bee is compared with the current food source *x*_*i*_ of its employed bee in the memory. If *ν*_*i*_≺_*η*_*r*_,*δP*_ *x*_*i*_, then the position *x*_*i*_ of the food source is changed to be *ν*_*i*_; otherwise *x*_*i*_ is kept as it is. Once all onlooker bees belonging to different employed bees finish their neighborhood search, the food sources of all the employed bees are compared according to ≺_*η*_*r*_,*δP*_ to obtain the global optimal food source of the second round of iterations. The second round of iterations ends. The third round of iterations will begin and so forth. At the end of each round of iterations, the PS-ABC approach needs to determine three problems. It firstly checks whether there exists any employed bee's food source which has not been improved continuously for LIMIT rounds of iterations and the food source is not the best one during LIMIT rounds of iterations. If yes, the employed bee has to abandon its current food source in the memory and become a scout to randomly search for a new food source according to expression (10). Then, the approach checks whether there exists any employed bee's food source which has not been improved continuously for LIMIT rounds of iterations and the food source is always the best one of all employed bees' food sources during LIMIT rounds of iterations. If yes, the approach stops iterative process and enters the next phase, which will be described in the following; if no, the PS-ABC approach will finally check whether the maximum number of iterations is reached or not. If it is met, the approach ends and returns the current global optimal food source (solution vector). Otherwise, the approach continues to the next round of iterations.

#### 4.4.4. Bayes Theorem Process of the PS-ABC Approach

**Figure 3:**
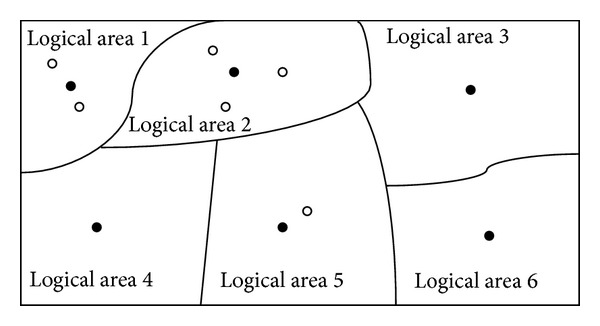
An example of logic area division of search space at some point.

As mentioned above, after each round of iterations, once the algorithm finds that there exists any employed bee's food source which has not been improved continuously for LIMIT rounds of iterations as well as the food source is always the best one of all employed bees' food sources during LIMIT rounds of iterations, PS-ABC stops iterative search process and enters the PS-ABC approach's Bayes theorem process proposed in this paper. It is obvious that the whole search space can be seen as FSN search areas by FSN employed bees logically at this moment. Each area has an employed bee as a master and has zero or several onlooker bees to follow its employed bee as slaves to search together for food sources within this area as shown in Figure 3.

The solid points represent the employed bees and the hollow points represent the onlooker bees in Figure 3. Here we take use of Figure 3 to illustrate the Bayes theorem process of the PS-ABC approach. We can regard each round of iterations as a randomized trial *E*. We define the search space as a sample space *S* of the randomized trial *E*. Let *A* be a random event: *A* = {a bee finds out the current global optimal food source (optimal solution vector)}. B1 (logical area 1), B2 (logical area 2),…, and B6 (logical area 6) are partitions of *S*. Assume that the PS-ABC has already finished *N* rounds of iterations before stopping. Besides, assume that the number of rounds for which the food source of each employed bee has been a current global optimal food source within the *N* rounds of iterations is *N*_*i*_. In other words, *N*_1_ + *N*_2_ + *N*_3_ + *N*_4_ + *N*_5_ + *N*_6_ = *N*. So, we can get the following conditional probability value:

(11) $$\begin{array}{c} P\left(A\;|\;B_{i}\right)=\frac{N_{i}}{N},\;i\in \left\{1,2,3,4,5,6\right\}. \end{array}$$

It represents the probability with which the bees in logical area *i* find out the current global optimal food source. The following probability value can also be obtained:

(12) $$\begin{array}{c} P\left(B_{i}\right)=\frac{{\text{Bee}}_{i}}{\text{Bee}}, \end{array}$$

where Bee_*i*_ represents the number of bees in logical area *i*. Bee is denoted to the total number of bees. Its reasonability lies in that each bee has the same ability in searching for the food sources by exploring and exploiting the whole search space. Thus we can obtain *P*(*B*_*i*_) in expression (12) according to geometric probability. This is also the theoretical basis for partitioning the whole search space. In terms of the proposed problem and our definition, we can calculate *P*(*B*_*i*_ | *A*) in order to make decisions in the next step according to the Bayes theorem as the following expression:

(13) $$\begin{array}{c} P\left(B_{i}\;|\;A\right)=\frac{P\left(B_{i}\right)P\left(A\;|\;B_{i}\right)}{\sum_{i=1}^{n}P\left(B_{i}\right)\cdot P\left(A\;|\;B_{i}\right)},\;i\in \left\{1,2,3,4,5,6\right\}. \end{array}$$

In these probability values obtained by calculating, the largest one *P*(*B*_*M*_ | *A*) is picked out. It represents that the final optimal food source is most likely to be in logical area *M*. At this point, all bees perform neighborhood search around the food source which the employed bee *M* is located at. The neighborhood search is repeatedly executed until the food source has not been improved for LIMIT rounds of neighborhood search continuously. At that moment, the PS-ABC approach returns the food source position. That is, the final solution vector is obtained. The algorithm ends.

Let us consider a problem in the proposed approach. We know that the solution vector of the proposed problem ought to be a sequence of integer numbers which denote the hosts in the resource pool. However, each food source of the PS-ABC is randomly initialized. What is more is that the expressions used by PS-ABC have coefficients which are limited between 0 and 1. Thus although the elements of each food source can be limited to an integer type in the implementation, the problem that the solution is not an integer still persists. To address this problem, we do not limit the elements to an integer type but employ the Smallest Position Value (SPV) rules presented in [13, 14]. In a nutshell, it is a rule which converts each food source's position vector given by the PS-ABC approach to a valid solution vector fit for the proposed problem. The process of applying the SPV rules into the proposed approach can be understood as follows. First, the hosts in the resource pool should be numbered from 0 to *m* − 1. Second, after the position of each food source is initialized randomly, all the elements of each position vector are sorted in ascending order and then are numbered from 0 to *n* − 1 as well as having a modulo operation of *m*. For instance, in a time window Δ*t*, there are four VMs to be migrated in a resource pool which has three hosts. If a position vector is (−1.21, 3.29, −0.12, 1.26), it will be converted to (0, 3, 1, 2) firstly and then to (0, 0, 1, 2). At this point, the solution vector is useful and meaningful. It represents that the target host of VM 0 is host 0. The target host of VM 1 is host 0. The target host of VM 2 is host 1. The target host of VM 3 is host 2. In the PS-ABC, all the food source position vectors refer to the vectors which the original vectors have been converted to according to the SPV rules.

In the proposed PS-ABC approach based on ABC, the bees are divided into two categories of employed bees and onlooker bees. And after an employed bee abandons its food source, it will become a scout. We know that each food source has an employed bee, which can be regarded as the master of a search group, the core of a logical area and keeps the position information of the current food source of this logical search group in the memory. What is more is that it can recruit onlooker bees with a certain probability by sharing its fitness information. It is well-known that this kind of swarm intelligence approaches such as ABC, particle swarm optimization (PSO), and ant colony optimization (ACO) has some common characteristics (virtues and deficiencies). On one hand, all of them are heuristic approaches and suitable for dealing with large-scale problems as well as having abilities in parallel or concurrent computing. On the other hand, they have some deficiencies due to their own characteristics such as premature and local-best solution. In PS-ABC, these onlooker bees as slaves are the main force of searching while the employed bees as masters should guarantee that the search focuses are rational and uniform.

In order to fully exert the virtues of ABC and eliminate its deficiencies, two important measures are adopted in the proposed PS-ABC approach. In the ABC approach, while onlookers and employed bees carry out the exploitation process in the search space, the scouts control the exploration process. However, at the beginning of PS-ABC, the initialization of food sources (i.e., the initial positions of employed bees) is also crucial for the next global search. The random initialization is not employed but the uniform random initialization method is presented and employed. It makes the PS-ABC approach have a better global exploration potential. It will get double results with half the effort. We know that in a robust search process, exploration and exploitation processes must be carried out together. Obviously, the core competency of ABC idea is exactly as follows: it can more easily make exploration and exploitation processes be carried out together by the cooperation of employed bees, onlooker bees, and scouts. However, its efficiency is not high enough since the classical ABC approach employs the roulette method, which does not take selective pressure into consideration, for onlooker bees' selection. In an iterative search algorithm, it should have better global search ability in the early iterations and it should have better local search and convergence ability in the later iterations. Thus, the selective pressure should change with the iterative process being keept on. More specifically, the onlooker bees should not be greedy and go to all employed bees as uniformly as possible in the early iterations in order to make the whole search space explored better and thus to avoid being trapped into local optimal food sources. In the later iterations, the onlooker bees gradually become greedy. That is, the employed bee with a better food source should have a larger probability value for onlooker bees' selection as well as to make the approach have high-efficient convergence ability and avoid premature. To achieve this goal, we have thought of simulated annealing (SA) idea [15, 16]. Further, by studying the SA idea, we have found the Boltzmann selection policy and introduced it into the proposed PS-ABC approach to thus achieve the self-adaptive selective pressure adjustment method. It makes the global exploration and local exploitation of employed bees, onlooker bees, and scouts more high-efficient and meaningful.

Starting from this intuition, the PS-ABC approach which aims at live VM migration policy gives consideration to the power saving optimization to achieve a more efficient cloud data center by designing and utilizing the ABC. During the course of the study, we have found that the search process of PS-ABC can be regarded as the repetitive randomized trials since it has the characters of randomized trials such as the nondeterminacy of results, the repeatability of trials, and the diversity of results and so forth. This lies in the iterative process of PS-ABC and its expressions with the random parameters. What is more is that in the PS-ABC approach, the whole search space can be divided into several logical search areas like some partitions of a sample space. This is peculiar to the ABC-based approach due to its design mode and search pattern. That is, an employed bee with some onlooker bees represents a logical search area. Its basis is that each bee has the same abilities in exploring and exploiting the search space. So, the whole search space can exactly be divided into several logical partitions (i.e., their union is the whole search space and their intersection is the empty set). At this point, the search of the global optimal food source can be seen as a random event in the sample space (search space).

Further, under this underground we can utilize the existing conditions and Bayes theorem to get the information about which logical area is most likely to have the global optimal solution. Upon obtaining this information, all bees can emphatically search this area and thus to quickly make the approach converge to the global optimal food source (solution vector). The triggering condition of Bayes theorem in PS-ABC has been described above. As a matter of fact, the design of its triggering condition has considered two aspects of problems at the same time. One is that if a food source has not been improved continuously for too many (LIMIT) rounds of iterations and it is always the optimal food source during these rounds of iterations, the food source will probably be the final global optimal solution or the global optimal solution is near it. However, we need to verify it. The core goal and idea of the Bayes theorem process is right here. Based on it, the triggering condition is set. The other is that the classical ABC approach has only considered the situation that a food source has not been improved continuously for too many (LIMIT) rounds of iterations and it is not the global optimal food source in each of these rounds of iterations. At that time, that food source will be abandoned by its employed bee. However, if the above food source is always the global optimal during these rounds of iterations, the classical ABC does not do any response. It is obvious that at this moment the algorithm should take action to respond to this phenomenon and thus to optimize the whole process. In addition, this is also the reason for that the round number of triggering condition is also set to LIMIT.

There are many parameters in the proposed PS-ABC approach. Most of them have great influences on PS-ABC. The maximum number MNI of iterations and the number FSN of employed bees (or onlooker bees) are two important parameters and their values are related to the efficiency and accuracy of PS-ABC. For instance, if the bees number FSN increases, the computation time (convergence time) of PS-ABC will increase and the possibility that the approach is converged to the global optimal solution will also increase. That is, the global search ability will strengthen. Moreover, the required iteration number MNI will decrease with FSN increasing. The maximum number MNI of iterations should be neither too small nor too large. Evidently, the size of the MNI value should make the current global optimal food source of each round of iterations of the PS-ABC approach exactly converge to the final global optimal food source as much as possible. Therefore, both the MNI value and the FSN value are the experience problems and need to perform lots of experiments to obtain fit values. In the proposed PS-ABC approach, FSN is set to 20 and MNI is set to 100 based on a large number of experiments.

Also, the controlling parameter LIMIT is another important value in PS-ABC since it has a direct impact on the implementation of the whole PS-ABC approach. It controls the transformation from an employed bee to a scout and controls when to enter the Bayes theorem process. Both of the two steps are crucial for PS-ABC. It is difficult for PS-ABC to assign to the controlling parameter LIMIT a fit value as it is related to several factors. Thus, it is an open problem which needs be studied further. In this paper, the LIMIT value is set to 20. Similarly, the *AL* value is an important controlling parameter proposed in the PS-ABC approach. Its value is an open problem. In this paper, it is set to 10. Besides, the initial temperature *T* and its attenuation factor *α* of Boltzmann selection policy are also important parameters. *T* should be initialized to an enough big value and it makes the corresponding algorithm have a stronger global search in the early iteration. Then *T* is gradually decreased with the iterative number increasing, which makes the search process gradually converge to an optimal solution in the later iteration. Thus, the values of *T* and *α* are important for Boltzmann selection idea. They should be set to the appropriate values as much as possible.

## 5. Evaluation

In this section, we have experimentally verified the proposed PS-ABC approach. The experiments have included evaluating the effect of power saving of PS-ABC and verifying the migration performance of PS-ABC migration policy on the failure rate of migration events. Besides, two auxiliary experiments have been conducted to evaluate a related parameter and get a better power management policy. In order to simulate a dynamic cloud data center, we have utilized an event driven simulator named CloudSim toolkit [17]. The CloudSim framework enables the accounting of the total power consumed by the system during the simulation period. In one word, the calculating of power consumption has been achieved by the CloudSim platform, which has provided a class including the methods getPower( ). CloudSim allows such simulation scenarios by supporting dynamic creation of different kinds of entities and can add and remove data center entities at run-time. This functionality has achieved simulating dynamic cloud environment where system components can join, fail, or leave the system randomly [17]. On CloudSim platform, we compare the proposed PS-ABC approach with the random migration policy and the optimal migration policy based on Genetic Algorithm (GA) by power consumption and the number of invalid VM migration. We have prepared four different kinds of experiments to evaluate and test the proposed approach. The results demonstrate that the proposed approach not only has a better power saving but also has a better migration performance compared to random migration policy and GA migration policy. Especially for a large number of migration events, the PS-ABC approach shows the stability and the better performance.

### 5.1. Experimental Scenarios

On CloudSim platform, a resource pool consisting of 100 hosts is created. These hosts have varying computing resource. 24 batches of virtual machine migration requests containing 13 requests randomly belonging to different hosts and with different resource requirements are created. The proposed PS-ABC module is invoked and fetches the resource information and state of the cloud resource pool periodically.

### 5.2. Comparison of GA, Random-Migration, and PS-ABC in Power Consumption

The experiment is designed for verifying the efficiency and availability of PS-ABC in power saving due to the placement selection of live VM migration during the operation of a cloud data center. In this scenario, we compare PS-ABC with Random-Migration and GA optimal migration policy by power consumption during the operation of the simulated cloud data center. In this experiment, Δ*t* is set as 600 seconds and the migration events of each batch are uniformly distributed within an hour, which includes 6Δ*t*s. Besides, the host's resource change rate is set as 1 time per half an hour.

**Figure 4:**
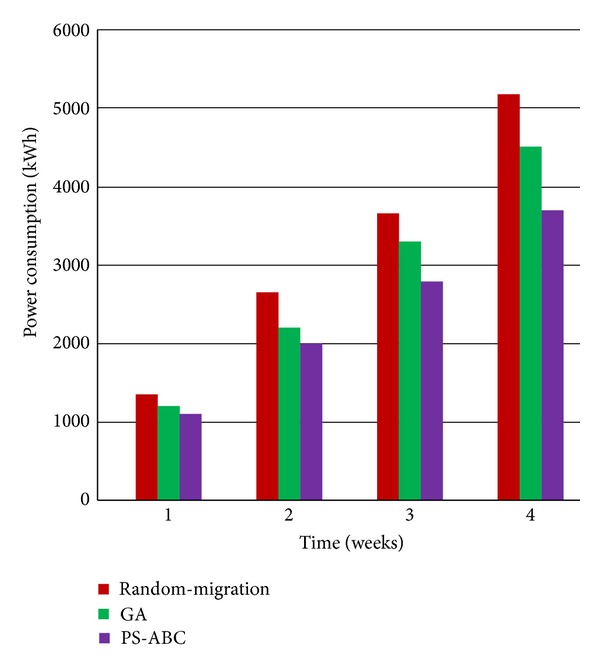
Comparison of GA, Random-Migration, and PS-ABC in power consumption.

As illustrated in Figure 4, at the end of each week, the cloud data center implementing PS-ABC consumes the least power than the cloud data center implementing GA and Random-Migration. The cloud data center implementing GA has a less power consumption than the cloud data center implementing Random-Migration. Moreover, it can also be seen that the cloud data center implementing PS-ABC has the least incremental power consumption between two weeks in view of the general trend. However, the incremental power consumption of Random-Migration has been increasing gradually between two weeks. This is because the Random-Migration policy is not sensitive enough to changes of the cloud environment and thus may cause more migration events or selects randomly an unreasonable target host in power consumption; with the passage of time, this phenomenon will be more and more obvious. For the GA policy, although it has a relatively less power consumption than Random-Migration, it is not so excellent as PS-ABC. It is well-known that GA is a widely used classical heuristic intelligent algorithm. However, compared with PS-ABC, it has some shortages. GA needs to code and decode each individual to represent a specific solution and enable the crossover and mutation operators. GA is not suitable for dealing with large-scale problems and it is easy to premature since its degree of parallelization and performance is not high enough. But for PS-ABC, it does not need the coding and decoding as well as genetic operators. From its algorithm and process, it can be seen that PS-ABC's degree of parallelization and performance is higher than that of GA. Accordingly, PS-ABC has a better power saving efficiency than GA naturally.

### 5.3. Comparison of the Number of Failures in VM Migration Events

**Figure 5:**
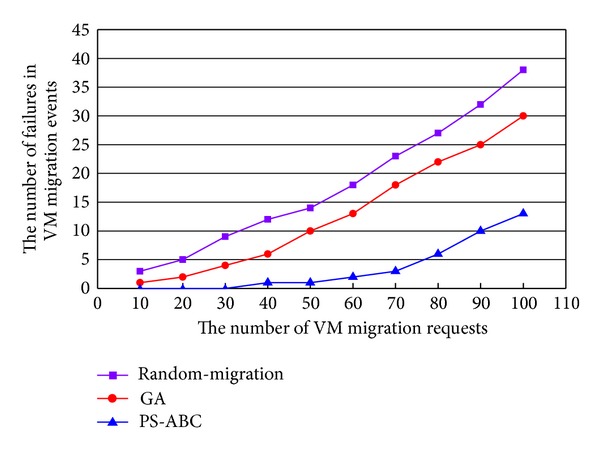
Comparison of the number of failures in VM migration events.

In the experiment scenario, the dynamic host failure is simulated by CloudSim by scheduling some host failure events and host shut down events to occur during the placement selection interval. These events have caused some failures in VM migration due to the nonavailability of the selected hosts for some VM migration requests. As illustrated in Figure 5, we have compared the PS-ABC approach with random migration and optimal migration implemented by GA. In the simulated cloud data center, with the increase of the number of VM migration requests, the cloud data center implementing Random-Migration and GA result in more number of invalid VM migrations, whereas the PS-ABC performs better in finding the fit hosts in the dynamic resource pool. This is because the memory data is outdated in the GA and Random-Migration. They cannot have an adjustment with the environment changed. Conversely, the PS-ABC policy which utilizes the Boltzmann selection policy and Bayes theorem is quite efficient in responding to the host failures during the interval as well as having a fit adjustment in a better manner by searching out the new available hosts that can meet the resource requirements of the VM migration requests. As described above, the proposed PS-ABC approach is designed to primarily meet performance requirements. On this basis, the power consumption is decreased as much as possible.

### 5.4. Comparison of GA and PS-ABC in Speed-Up Ratio

**Figure 6:**
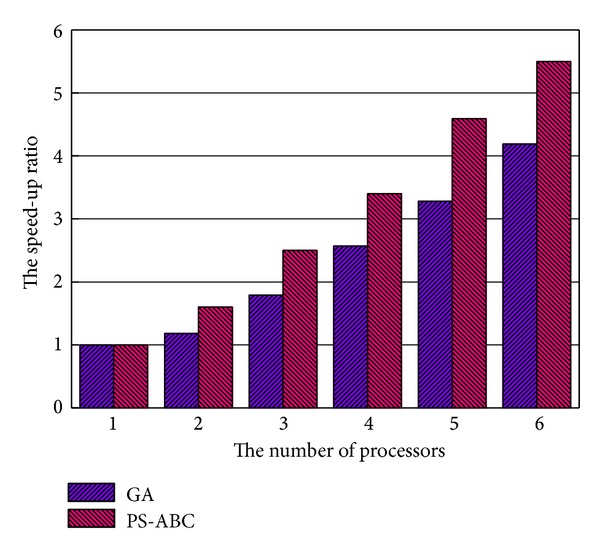
Comparison of GA and PS-ABC in speed-up ratio.

In this experimental scenario, we will evaluate the speed-up ratios of GA and PS-ABC with the number of processors increasing. The speed-up ratio *S*_*p*_ can be expressed as follows:

(14) $$\begin{array}{c} S_{p}=\frac{T_{1}}{T_{p}}, \end{array}$$

where *T*_1_ is the processing time of a task under the condition of single processor and *T*_*p*_ refers to the running time of the same task under the condition of *p* processors. Thus, the speed-up ratio *S*_*p*_ can be used to measure the performance and effect of program parallelization. As shown in Figure 6, the experiment shows the speed-up ratios of PS-ABC and GA under these conditions of different number of processors. With the increase of the number of processors, the speed-up ratio of PS-ABC is always larger than that of GA. The proposed PS-ABC approach is more close to the linear speed-up. For the PS-ABC approach, it can see the whole search space as multiple independent search subspace logically. Each of these subspaces performs its own search independently and then the employed bees, as the representatives of these logic search subspaces, share their information uniformly. The mechanism and process of PS-ABC is easy to parallel processing. Whereas for GA, it does not have these features and advantages. Its mechanism is suitable for centralized processing relatively. Thus, the performance and efficiency of PS-ABC are more excellent than those of GA. In particular, in cloud data centers, the PS-ABC approach is faster and more efficient.

### 5.5. Comparison of the Incremental Power Consumption in a Cloud Data Center with Varying Percentage of Load

**Figure 7:**
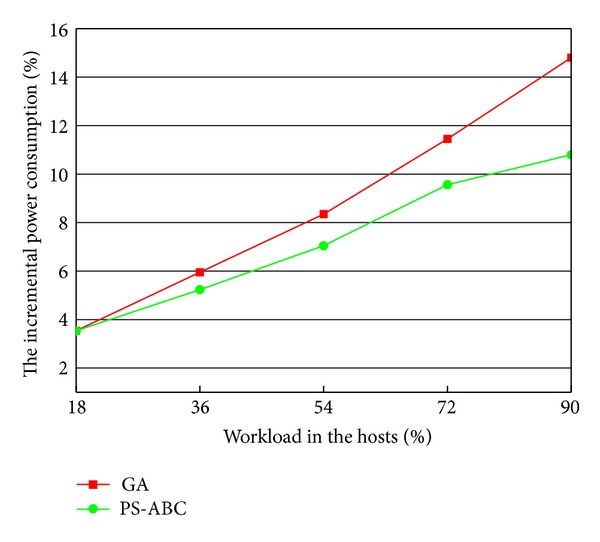
Comparison of the incremental power consumption in the cloud data center with varying percentage of load.

In this experimental scenario, we compare the incremental power consumption of GA and PS-ABC in a cloud data center with varying percentage of load. The load mentioned refers to the load of the whole cloud data center and is not the load of some physical host. The varying percentage of load means that this experiment has been done in the same cloud data center with a different load. As illustrated in Figure 7, with the increase of the percentage of load in the cloud data center, the incremental power consumption of PS-ABC is less than that of GA. We know that, if the load in the hosts is heavier, the scale of the problem to be solved will be larger. That is, after a time window Δ*t*, the number of VMs which need to be migrated is larger. Compared with GA, the PS-ABC approach is more suitable for solving the relatively larger-scale problems. Its final returned optimal solution vector is more accurate. Therefore, PS-ABC is more efficient power-saving migration policy from a long-term view for the cloud environment with a heavy load.

### 5.6. Trade-Off between Power Saving and Performance Fulfillment

**Figure 8:**
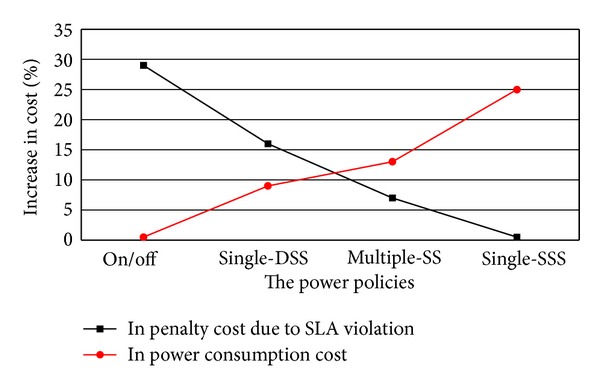
The trade-off between power and performance with different power management policies.

The experimental scenario is conducted to find the optimal power management policy which balances the benefits due to power saving and performance fulfillment. Since live VM migration events are time critical VM requests and the cloud service provider should meet strict Service Level Agreements (SLA) compliance, any violations in SLA in terms of performance loss of VMs will result in penalty cost on the provider of a cloud data center. The performance loss mentioned may be caused by power saving, live VM migration, network bandwidth, throughput, and so forth. However, this paper has not focused on these problems. This experiment is designed to only find out a better power management policy suitable for the cloud environment implementing the proposed PS-ABC to have a trade-off between the benefit of power saving and penalty cost due to SLA violation. There are four different policies to be formulated. The first policy is On/Off policy, wherein all idle hosts are switched off. It can be seen that the policy gives the best power saving, but it causes the high penalty cost obviously. The single-DSS policy is the second policy, wherein all idle hosts are switched to deep sleep state. It results in an increase in the power consumption cost, but the penalty cost is reduced significantly. The third policy is single-SSS, wherein all idle hosts are switched to shallow sleep state. There is no penalty cost as SLA violation is absent in this policy. However, the enormous increase in the power consumption cost is caused. The multiple-SS is the fourth policy, wherein some of the idle hosts are kept in deep sleep and others are kept in shadow sleep state according to a short-term prediction technology. From its meaning and as shown in Figure 8, it can be known that the multiple-SS policy gives the optimal cost trade-off relatively.

## 6. Conclusion and Future Work

In this paper, a novel placement selection policy of live VM migration PS-ABC is proposed and we give its algorithm, implementation, and evaluation. It is based on the improved ABC-based approach which employs the Boltzmann selection idea and Bayes theorem. In the improved ABC-based approach, we introduce the binary search idea into the classic ABC approach to achieve the uniform random initialization and thus to make the whole PS-ABC approach have a better global search potential and capacity at the very beginning. In the proposed PS-ABC approach based on ABC approach, we have employed *AL Times of Attempts* policy of neighbourhood search in order to improve the efficiency of searching without too much additional overhead while making the first fitness function of performance requirements become a constraint condition and thus to be consistent with the real needs. Also, PS-ABC does not employ the roulette wheel selection for onlooker bees' selection but utilizes the Boltzmann selection policy of simulated annealing idea to make the proposed approach have the self-adaptive selective pressure adjustment and thus to achieve having the better global exploration ability in the early stage and the better exploitation ability in the later stage. What is more is that we have introduced the Bayes theorem into the proposed PS-ABC approach. A sample space and its all elements are presented and formalized. By calculating probability values and using them, the PS-ABC approach can be faster converged to the global optimal solution vector. It not only makes the PS-ABC approach have abilities in fast convergence in some cases but also fills in the gap of the classical ABC approach. It has shown that the proposed PS-ABC approach has the strong mathematical basis.

PS-ABC achieves the high-efficiency of power consumption and the stability of requirement performance. It minimizes not only the incremental power consumption of a cloud data center but also the number of failure in VM migration events relatively. It aims at achieving the better power conservation during the long-term operation of a cloud data center and protects the performance of VM running. In the proposed PS-ABC approach, there are some open problems which need to be studied further and experience problems which need many experiments to gradually get a better solution. The maximum number MNI of iterations and the number FSN of employed bees (or onlooker bees) as well as the *T* value of Boltzmann selection policy are the experience problems and we need to perform several experiments to obtain the fit values to thus make the PS-ABC approach efficient and feasible. The controlling parameters LIMIT and *AL* of our approach as well as the attenuation factor *α* value of Boltzmann selection policy are the open problems which need to be further widely researched. In this paper, all the parameters are set to the fit values, respectively.

To evaluate the PS-ABC approach, we have conducted several experiments on the CloudSim platform. Firstly, in the comparison experiment of GA, Random-Migration, and PS-ABC in power consumption, the PS-ABC approach has the least incremental power consumption during the long-term operation of the cloud data center. Secondly, in the comparison experiment of the number of failures in VM migration events, the PS-ABC approach has the least number of failures in VM migration events than that of random migration and GA optimal migration. Thirdly, in the comparison experiment of GA and PS-ABC in Speed-up Ratio, the result has shown that the proposed PS-ABC approach has the better speed-up ratio and correspondingly it has the better efficiency compared with the GA migration policy. Finally, in the comparison experiment of the incremental power consumption in the cloud data center with varying percentage of load, the result has shown that the PS-ABC approach is more efficient in power saving than random migration and GA optimal migration for the cloud data center with a heavy load during the long-term operation of a cloud data center. In order to balance the relationship between the power conservation and performance fulfillment, we have also performed an experiment to find out the better power management policy and proved that the multiple-SS policy is the best selection. The final experimental results show that PS-ABC is an effective placement selection policy of live VM migration for power conservation.

Aiming to further improve the performance of PS-ABC, we plan to study the robustness of PS-ABC in the next step work. PS-ABC should have abilities in dealing with some sudden matters and be combined with the mechanism of live VM migration for achieving a more efficient hybrid management. Besides, in this paper, PS-ABC is used for the placement selection of live VM migration in LAN. In the future, we will extend PS-ABC in WAN. In the next work and experiments, the experience problems and the open problems presented in this paper will also be researched further. If possible, we will research and implement the proposed PS-ABC approach in a real cloud computing environment as well as evaluating its performance and efficiency.
